# Supplementary material for: Content-rich biological network constructed by mining PubMed abstracts
Source: BMC Bioinformatics. 2004 Oct 8;5:147. doi: 10.1186/1471-2105-5-147 (PMC528731; doi:10.1186/1471-2105-5-147)
Supplement: Additional File 5 — The original Chilibot query results of the term "long-term potentiation (LTP)" and 22 other terms, limiting the latest references analyzed to the years 1990, 1995, 2000, and 2004. [file 1471-2105-5-147-S5.bz2 › chilibotAdditionalFile5/ltp2004/html/PLC_CREB.html]

 


 **PLC** and **CREB** 
  
Found 3 abstracts in PubMed,  **3 abstracts were retrieved and analyzed**.  


---

 Search Google  |
 PDF files only 
|  EDU domain only 

---

- Biochem Biophys Res Commun, 2002   **Regulation of cyclic AMP dependent response element binding protein **CREB** by the nociceptin orphanin FQ in human dopaminergic SH SY5Y cells.** .
  Nociceptin orphanin FQ N OFQ , an endogenous ligand for opioid receptor like ORL1 receptor, transduces signaling cascades implicated in MAPK, PKC, **PLC**, and calcium, etc.
  This study was designed to investigate the intracellular signaling mechanism of N OFQ in human dopaminergic neuroblastoma SH SY5Y cells.
  N OFQ rapidly induced the phosphorylation of **CREB**, which was significantly suppressed by pretreatment of PKA inhibitor, but not by MAPK inhibitors.
  It also time dependently increased the phosphorylation of MAPK, which was proven as ERKs.
  . . . it did not affect the PI3K activity.
  Interestingly, KT5720, a specific inhibitor of PKA, markedly suppressed the phosphorylation of MAPK by N OFQ in SH SY5Y cells.
  Furthermore, BAPTA AM, an intracellular chelator of Ca 2ion , completely abolished the phosphorylation of **CREB** as well as MAPK in N OFQ treated SH SY5Y cells.
  Taken together, these results suggest that N OFQ independently induces the activation of **CREB** prior to MAPK phosphorylation, which was also modulated by PKA.
  Furthermore, Ca 2ion related signaling implicates in the phosphorylation processes of **CREB** and MAPK simultaneously.

  - Mol Neurobiol< MedlineTA>Mol Neurobiol,   **Neuronal signaling systems and ethanol dependence.** .
    In recent years there have been remarkable developments toward the understanding of the molecular and or cellular changes in the neuronal second messenger pathways during ethanol dependence.
    In general, it is believed that the cyclic adenosine monophosphate cAMP and the phosphoinositide PI signal transduction pathways may be the intracellular targets that mediate the action of ethanol and ultimately contribute to the molecular events involved in the development of ethanol tolerance and dependence.
    Several laboratories have demonstrated that acute ethanol exposure increases.
    . . . protracted ethanol exposure decreases, agonist stimulated adenylate cyclase activity in a variety of cell systems, including the rodent brain.
    Recent studies indicate that various postreceptor events of the cAMP signal transduction cascade i.e.
    , Gs protein, protein kinase A PKA , and cAMP responsive element binding protein **CREB** in the rodent brain are also modulated by chronic ethanol exposure.
    The PI signal transduction cascade represents another important second messenger system that is modulated by both acute and chronic ethanol exposure in a variety of cell systems.
    It has been shown that protracted ethanol exposure significantly decreases phospholipase C **PLC** activity in the cerebral cortex of mice and rats.
    The decreased **PLC** activity during chronic ethanol exposure may be caused by a decrease in the protein levels of the **PLC** beta 1 isozyme but not of **PLC** delta 1 or **PLC** gamma 1 isozymes in the rat cerebral cortex.
    Protein kinase C PKC , which is a key step in the PI signaling cascade, has been shown to be altered in a variety of cell systems by acute or chronic ethanol exposure.
    It appears from the literature that PKC plays an important role in the modulation of the function of various neurotransmitter receptors e.g., gamma aminobutyrate type A GABAA , N methyl D aspartate NMDA , serotonin2A 5 HT2A , and 5 HT2C, and muscarinic m1 receptors resulting from ethanol exposure.
    The findings described in this review article indicate that neuronal signaling proteins represent a molecular locus for the action of ethanol and are possibly involved in the neuro adaptational mechanisms to protracted ethanol exposure.
    These findings support the notion that alterations in the cAMP and the PI signaling cascades during chronic ethanol exposure could be the critical molecular events associated with the development of ethanol dependence.
